# Comparison of the Prognostic Performance of Various Machine Learning Models in Patients with Acute Myocardial Infarction: Results from the COREA-AMI Registry

**DOI:** 10.3390/medicina61101783

**Published:** 2025-10-02

**Authors:** Ji-Hoon Jung, Kyusup Lee, Kiyuk Chang, Youngkeun Ahn, Sung-Ho Her, Sangin Lee

**Affiliations:** 1 Department of Information and Statistics, Chungnam National University, Daejeon 34134, Republic of Korea; jihoon.jung@kitox.re.kr; 2 Korea Institute of Toxicology, Daejeon 34114, Republic of Korea; 3 Department of Cardiology, Daejeon St. Mary’s Hospital, College of Medicine, The Catholic University of Korea, Seoul 34943, Republic of Korea; 4 Department of Cardiology, Seoul St. Mary’s Hospital, College of Medicine, The Catholic University of Korea, Seoul 06591, Republic of Korea; kiyuk@catholic.ac.kr; 5 Department of Cardiology, Chonnam National University Hospital, Gwangju 61469, Republic of Korea; cecilyk@hanmail.net; 6 Department of Cardiology, St. Vincent’s Hospital, College of Medicine, The Catholic University of Korea, Seoul 16247, Republic of Korea; hhhsungho@naver.com

**Keywords:** acute myocardial infarction, machine learning, prognosis, predictors, random forest

## Abstract

*Background and Objectives*: To date, several machine learning (ML) prognostic prediction models have been investigated for patients with acute myocardial infarction (AMI). However, few studies have compared the prognostic performance of ML techniques in AMI patients who underwent percutaneous coronary intervention (PCI). We sought to compare the prognostic performance among various machine learning techniques to determine which one showed the best prediction ability. *Materials and Methods*: Using data from the large, multicenter COREA-AMI registry, this study analyzed 10,172 patients to predict major adverse cardiac events (MACEs) at 1 and 5 years. MACE was defined as a composite of cardiac death, myocardial infarction, or cerebrovascular accident. *Results*: Compared with the four other ML techniques and traditional logistic regression, the random forest (RF) model consistently demonstrated the highest predictive performance. At 5 years, the RF model achieved a superior area under the curve (AUC) of 0.822, an accuracy of 0.804, and an F1 score of 0.870. To ensure clinical interpretability, a SHapley Additive exPlanations analysis was performed on the RF model. It identified key independent predictors for MACEs. The top nonmodifiable predictors included age, renal function, and left ventricular ejection fraction, whereas modifiable risk factors included dual antiplatelet therapy, statin therapy, angiotensin-converting enzyme inhibitor/angiotensin receptor blocker therapy, and adherence to these optimal medical therapy. *Conclusions*: In this real-world patient cohort, the RF model provided modest improvements in long-term risk stratification, and our findings highlight the continuing importance of guideline-directed medical therapy in determining patient prognosis.

## 1. Introduction

In Korea, the prevalence of acute myocardial infarction (AMI) has increased over the past 10 years [1]. AMI has consistently been associated with poorer clinical outcomes compared with a first event [2,3]. Therefore, secondary prevention with guideline-derived optimal medical therapy is important for reducing the risk of recurrent adverse cardiovascular events [4].

Acute myocardial infarction (AMI) remains a leading cause of death globally [5,6], making accurate prognostic evaluation essential for risk stratification and the selection of optimal treatment strategies for patients. Historically, traditional statistical methods such as logistic regression analysis have served as the cornerstone for evaluating prognostic predictors in this population. However, these models are often limited in their ability to capture complex, nonlinear relationships. In recent years, machine learning (ML) techniques have emerged as powerful tools for developing predictive models capable of identifying complex and nonlinear patterns within large datasets of patients with coronary artery disease [7]. Several studies have demonstrated the clinical utility of ML in predicting in-hospital and midterm outcomes after AMI [8,9].

However, data remain limited regarding the long-term comparative performance of different ML algorithms, especially in large real-world cohorts of AMI patients treated with percutaneous coronary intervention (PCI). Furthermore, a significant barrier to the clinical adoption of many ML models is their ‘black box’ nature. For clinicians to trust and act upon these predictions, it is crucial that the models be not only accurate but also interpretable.

Therefore, we aimed to compare the long-term prognostic performance of several ML techniques, including traditional logistic regression, and to identify major clinical predictors using interpretable ML-based approaches.

## 2. Materials and Methods

### 2.1. Study Design and Population

The CardiOvascular Risk and identification of potential high-risk populations in the Acute Myocardial Infarction (COREA-AMI) registry is a retrospectively collected, prospective, multicenter, all-comer cohort study designed to evaluate clinical outcomes and risk factors in patients with AMI. The registry was established and is primarily coordinated by the Catholic University of Korea and its affiliated hospitals in collaboration with multiple tertiary referral centers across the nation under the support of the Korean Society of Interventional Cardiology. A total of 10,719 patients who underwent PCI between April 2004 and April 2015 were enrolled in the registry.

**Figure 1:**
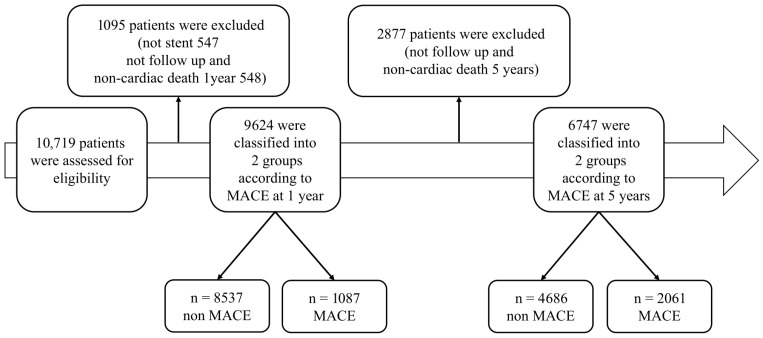
**Study flow chart.** MACE, major adverse cardiac event.

Among the 10,719 patients, we included consecutive patients who were treated with drug-eluting stents (DESs) during the index PCI for the current analysis. Patients who did not undergo stent implantation or who were lost to follow-up were excluded (Figure 1). To reduce potential multicollinearity and ensure model robustness, we excluded redundant variables that conveyed overlapping information (e.g., body mass index [BMI] instead of height and weight and age instead of date of birth). In addition, variables of in-hospital adverse events (such as cardiogenic shock, use of inotropes, dialysis, transfusion, and acute heart failure) were not included, as they represent in-hospital outcomes rather than predictors. Finally, a total of 64 variables were selected for analysis (Table 1), including 7 demographic variables, 14 related to past medical history, 14 laboratory and echocardiographic variables, 7 discharge medication variables, and 22 procedural variables.

### 2.2. Ethics Statement

The study protocol of the COREA-AMI registry was conducted in accordance with the Declaration of Helsinki and was approved by the Institutional Review Boards (IRBs) of all the participating centers (IRB approval code: XC13RIMI0060K) on 25 July 2013. It was also registered at ClinicalTrials.gov (NCT02385682). Written informed consent was not needed, as this study was approved by the Institutional Review Boards in accordance with national regulations for retrospective observational studies.

### 2.3. Study Outcomes

The primary endpoint was the rate of major adverse cardiac events (MACEs) at 5 years, defined as a composite of cardiac death, MI and cerebrovascular accident. The secondary endpoint was the occurrence of MACEs at 1 year. All clinical events were recorded at each participating hospital and were centrally adjudicated by an independent panel of clinicians. To assess long-term prognostic performance, we selected 5-year MACEs as the primary endpoint. In addition, we analyzed 1-year MACEs to examine differences in model performance and predictor contributions between the 1-year and 5-year outcomes. This approach also enabled us to evaluate how the relevance of specific predictors evolves over time.

### 2.4. Statistical Method and Machine Learning Model

Continuous variables are presented as the mean values with standard deviations, and categorical variables are presented as frequencies with percentages. The registry originally included 121 variables, including past disease history, laboratory findings, procedural variables, medications and demographic characteristics. As described in Section 2.1, after excluding redundant variables and those reflecting in-hospital events, a total of 64 variables were finalized for model construction and subsequent analysis.

Missing data were assumed to be Missing at Random (MAR) and were addressed using multiple imputation based on the Markov Chain Monte Carlo (MCMC) method implemented through the PROC MI procedure in SAS (version 9.4), generating five imputed datasets under the assumption of multivariate normality [10,11,12]. The proportion of missing values varied across variables, ranging from 0.3% to 38.4%, with HbA1C showing the highest rate (38.4%) and most variables having less than 10% missing values. Missingness was observed in body mass index (BMI, 3.9%), systolic blood pressure (SBP, 1.1%), diastolic blood pressure (DBP, 1.2%), heart rate (HR, 1.2%), left ventricular ejection fraction (LVEF, 7.0%), white blood cell (WBC, 0.5%), neutrophil (0.5%), hemoglobin (Hb, 0.3%), platelet (0.4%), glycated Hb (HbA1c, 38.4%), total cholesterol (TC, 7.1%), triglyceride (TG, 7.6%), high-density lipoprotein cholesterol (HDL-C, 7.7%), low-density lipoprotein cholesterol (LDL-C, 10.1%), high-sensitivity C-reactive protein (hsCRP, 16.5%), creatinine (Cr, 0.3%), estimated glomerular filtration rate (eGFR, 0.3%), and creatine kinase–myocardial band (CK-MB, 0.7%), whereas all other variables had no missing values.

The primary outcome, MACE, was defined as a binary variable (0 = no event, 1 = event). Given the binary nature of the outcome and the potential for class imbalance, the dataset was split into training (70%) and testing (30%) subsets using stratified sampling to maintain the proportion of MACE cases [13,14]. To ensure robustness against sampling bias and random variability, the entire process of data splitting, model training with hyperparameter tuning, and evaluation was repeated 100 times, and the average performance metrics across these iterations were used for final comparison [15].

Prediction models were developed and evaluated using five machine learning algorithms: traditional logistic regression, naïve Bayes (NB), elastic net regression, support vector machine (SVM), and random forest (RF) [16,17,18,19,20]. For each model, hyperparameter tuning was conducted using 5-fold cross-validation within the training dataset. For elastic net, the optimal alpha and lambda values were determined using a grid search. For the SVM, a radial basis function (RBF) kernel was applied, with both the cost (C) and the gamma parameters tuned. The number of trees and maximum depth were optimized for the random forest models. For models sensitive to class imbalance (e.g., logistic regression and SVM), class weights were adjusted accordingly [21,22,23,24,25].

The prediction performance of each model was evaluated using five standard metrics: accuracy, precision, recall, F1 score (the harmonic mean of precision and recall), and the area under the receiver operating characteristic curve (AUC) [26,27]. These metrics provide a comprehensive view of classification effectiveness, particularly in the context of imbalanced outcomes. To enhance model interpretability and identify key prognostic predictors of MACEs, we conducted a SHapley Additive exPlanations (SHAP) analysis on the best-performing model, namely, the random forest model [28,29]. SHAP values quantify the marginal contribution of each variable to the model’s predictions.

All the statistical analyses and modeling procedures were conducted using R (version 4.3.0) and Python (version 3.6.5). Key libraries included scikit-learn, xgboost, and SHAP in Python and caret, glmnet, and randomForest in R [14,30,31,32,33]. Random seeds were fixed to ensure reproducibility across iterations.

## 3. Results

### 3.1. Baseline Characteristics and Clinical Outcomes

From the COREA-AMI registry (*n* = 10,719 patients), a total of 6747 patients were included in the 5-year analysis, among whom 2061 (30.5%) experienced MACEs. For the 1-year analysis, 9624 patients were included, and 1087 patients (11.3%) experienced MACEs (Figure 1).

As shown in Table 1, patients who experienced MACEs at both 1 and 5 years were older and had a higher incidence of comorbidities such as hypertension, diabetes mellitus, atrial fibrillation, chronic renal disease, chronic obstructive pulmonary disease and malignancy. Procedural complexity, including left main PCI and long stenting, was also more frequent in the MACE group, whereas the use of intravascular ultrasound (IVUS) was less common. In contrast, lesion characteristics such as bifurcation, ostial, or chronic total occlusion lesions did not significantly differ between the groups. These trends were consistent across both time points.

### 3.2. Comparative Performance of Machine Learning Models

**Figure 2:**
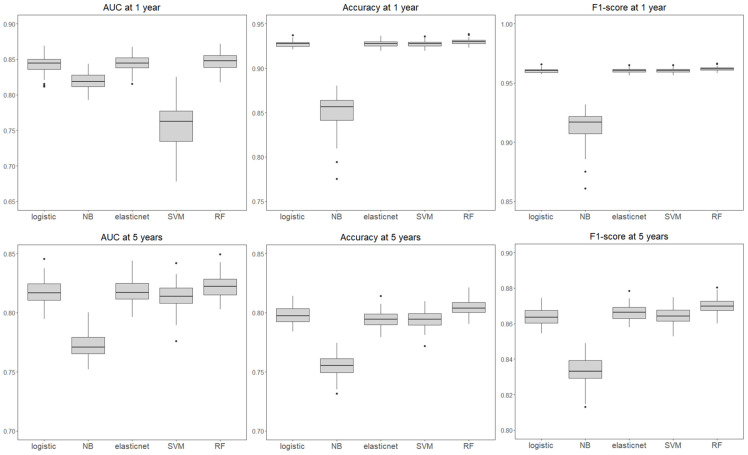
**Box plots of the performances of different models.** AUC, area under the curve; NB, naïve Bayes; elastic net, elastic net regression; SVM, support vector machine; RF, random forest.

We compared the performance of four ML models—RF, SVM, elastic net, and NB—with that of traditional logistic regression for predicting MACEs at 1 and 5 years (Table 2 and Figure 2). The RF model consistently achieved the highest predictive performance across all the evaluation metrics, including the AUC, accuracy, and F1 score. At 5 years, the RF model achieved an AUC of 0.822, an accuracy of 0.804, and an F1 score of 0.870, which were superior to those of logistic regression (AUC 0.817, accuracy 0.798, F1 score 0.864). At 1 year, the performance gap became more prominent, with RF showing an AUC of 0.847, an accuracy of 0.930, and an F1 score of 0.962, compared with logistic regression (AUC of 0.843, accuracy of 0.927, and F1 score of 0.960). This superiority of the RF model was consistently observed in both the 1-year and the 5-year predictions.

Among alternative algorithms, elastic net and SVM showed comparable results to logistic regression, whereas the NB model demonstrated the lowest predictive performance, particularly for 5-year MACE (AUC 0.772; accuracy 0.755; F1 score 0.834).

### 3.3. Prognostic Predictors and SHAP Analysis

**Figure 3:**
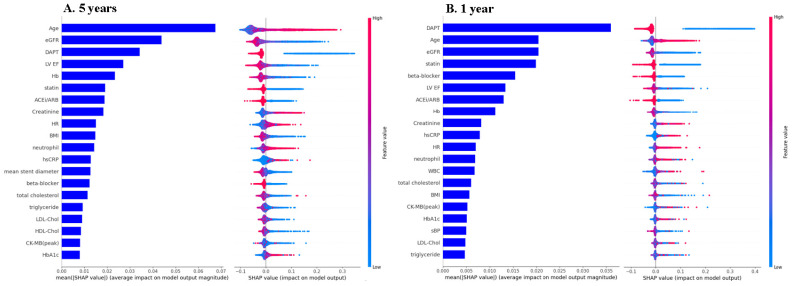
**SHAP plot. (A). 5 years; (B). 1 year.** eGFR, estimated glomerular filtration rate; DAPT, dual antiplatelet therapy; LV EF, left ventricular ejection fraction; Hb, hemoglobin; ACEi/ARB, angiotensin-converting enzyme inhibitor/angiotensin receptor blocker; HR, heart rate; BMI, body mass index; hsCRP, high-sensitivity C-reactive protein; LDL-Chol, low-density lipoprotein cholesterol; HDL-Chol, high-density lipoprotein cholesterol; CK-MB, creatine kinase–myocardial band; HbA1c, glycated hemoglobin.

We further clarified the most influential predictors using SHAP analysis based on the RF model. The top 20 predictors for MACEs at 1 and 5 years according to the mean absolute SHAP value are shown in Figure 3. At 5 years, the top 5 predictors were age, estimated glomerular filtration rate (eGFR), dual antiplatelet therapy (DAPT), left ventricular ejection fraction (LV EF), and hemoglobin at 5 years. Similarly, at 1 year, DAPT was the most important predictor, followed by age, eGFR, statin use and beta-blocker use. The SHAP value plot revealed that older age, impaired renal function, the absence of DAPT, reduced LV EF, and anemia were associated with increased long-term adverse events. Notably, DAPT and statin use ranked higher at 1 year, highlighting the importance of optimal medical therapy for early prognosis.

## 4. Discussion

The major findings from the present analysis are as follows: (1) the incidence rates of 1-year and 5-year MACEs in our study are comparable to those reported in previous studies; (2) the RF model demonstrated slightly better prognostic performance compared with traditional statistical methods (logistic regression analysis); and (3) SHAP analysis enabled us to identify the independent predictors for both 1-year and 5-year MACEs.

At 5 years, MACEs occurred in 2061 patients (30.5%). At 1 year, MACEs occurred in 1087 patients (11.3%). These findings are consistent with those of prior studies. For instance, Kim Y et al. reported a 1-year adverse event rate (including all-cause mortality, MI, and any revascularization) of 12.3% in a national Korea AMI registry [34]. Similarly, a nationwide cohort study of 35,972 AMI patients who underwent PCI reported 4-year event rates of all-cause death and coronary revascularization ranging from 26.4% to 28.5% [4].

Although several studies have investigated ML-based prognostic models in AMI patients, many have been limited by their focus on all-cause mortality, in-hospital outcomes, or short-term follow-up [9,35,36]. In contrast, a key strength of our study is its focus on a specific composite of MACEs: cardiac death, MI, and cerebrovascular accidents. This approach offers a more direct assessment of long-term cardiovascular prognosis, as the endpoint is not influenced by noncardiac causes of death or by discretionary decisions such as revascularization. Furthermore, by evaluating risk for both 1-year and 5-year clinical outcomes, our study provides not only a more comprehensive perspective on long-term prognosis but also an understanding of the temporal dynamics of predictors. This large, multicenter, real-world nature of our PCI cohort enhances the generalizability of these findings, underscoring their relevance in routine clinical practice.

The RF model exhibited the highest predictive performance for both 1-year and 5-year MACE prediction in our analysis. Although many ML studies in clinical research report that model performance is primarily based on the AUC, this metric can be misleading in datasets with imbalanced outcomes. This is a common challenge in predicting clinical events such as MACEs, where the number of nonevents significantly outweighs the number of events. Recent studies have increasingly recognized this limitation, highlighting the need for more robust evaluation metrics [37,38].

Consistent with other real-world AMI registries, our study population exhibited significantly imbalanced outcomes, with a MACE incidence of 11.3% at 1 year and 30.5% at 5 years. In evaluating model performance under such imbalance, precision and recall are particularly important metrics, while AUC and accuracy also provide complementary information [26]. The precision (also called the positive predictive value) is the fraction of relevant instances among the retrieved instances, whereas the recall (also known as the sensitivity) is the fraction of relevant instances that were retrieved [39]. Therefore, the F1 score, which is derived from the harmonic mean of precision and recall, is particularly meaningful in our study [40]. We demonstrated that the F1 score of the RF model was the highest among all the ML techniques and logistic regression analyses.

Our study also provides valuable insights through SHAP analysis. A prior study by Oliveira M et al. employed SHAP to predict in-hospital mortality and identified variables such as cardiac dysrhythmia, glucose, cardiogenic shock, and renal failure [36]. However, many of these variables likely reflect effects of clinical deterioration rather than independent risk factors, thus limiting their use in long-term prognostic models. In contrast, our study excluded in-hospital complication variables such as cardiogenic shock, inotrope use, dialysis, transfusion, and acute heart failure, recognizing that these factors are not independent predictors but rather clinical consequences of deterioration status. As our goal was to identify modifiable predictors of long-term outcomes, we focused on baseline or discharge-level predictors that can inform long-term management strategies, particularly those related to secondary prevention.

Recently, Yang Y et al. established ML models for short-term (1 year) and long-term (5 years or 10 years) mortality prediction for AMI using SHAP analysis [37]. Using 4173 patients, the dataset was allocated into three groups (1-year group: 3626; 5-year group: 2102; and 10-year group: 721). Although our study shares some common findings with that study, a notable divergence emerges when we compare the top predictors identified by our SHAP analysis, such as ‘operation time’ or ‘apolipoprotein A’. These differences likely reflect the distinct endpoints and patient populations of the two studies. This study focused on all-cause mortality in a broader AMI population.

In contrast, our study was designed to predict a composite of MACEs within a well-defined cohort of post-PCI patients. Notably, the high ranking of predictors such as DAPT, statin, and LV EF in our analysis strongly aligns with current evidence-based clinical guidelines. This alignment underscores the clinical relevance and practical application of our model’s findings for optimizing patient care in a routine post-PCI setting.

Using this approach, we identified clinically relevant predictors of long-term outcomes in AMI patients. Among the modifiable factors, optimal medical therapy, such as dual antiplatelet therapy (DAPT), statins, and angiotensin-converting enzyme (ACE) inhibitors or angiotensin receptor blockers (ARBs), was consistently associated with a lower risk of MACEs. Beta-blockers also emerged as a predictor, although with relatively weaker importance. These findings are consistent with current clinical guidelines, which recommend extended DAPT and statin therapy for secondary prevention in AMI patients (class I recommendation) [41,42,43]. However, the long-term benefits of beta-blocker maintenance therapy remain a subject of ongoing debate, particularly in patients without left ventricular dysfunction or heart failure [44,45].

Interestingly, nonmodifiable predictors, such as age, renal function, hemoglobin level, and LV EF, ranked highest in terms of predictive power for both 1-year and 5-year MACEs. In contrast, the procedural variables (e.g., total stent length and mean stent diameter) had lower SHAP values, indicating a weaker prognostic influence. Although this may suggest that long-term prognosis is more strongly influenced by patient characteristics and medical therapy than by procedural details, it is important not to overinterpret these findings. Key procedural aspects, such as stent optimization or intravascular imaging use, which were not fully captured in our dataset, may play a significant role. Future studies incorporating these variables could better delineate the impact of procedural quality on long-term outcomes.

Our analysis has the following potential limitations: (1) This study was an observational registry study with inherent selection bias. (2) To accurately assess the prognostic performance of ML techniques for cardiovascular events, we excluded patients who died from noncardiac causes, which could limit the generalizability of our findings. (3) As the study population included only patients from South Korea, it is uncertain whether our findings can be directly applied to other ethnic groups. (4) The registry enrolled patients over a long period (2004–2015), during which time significant evolutions in stent technology and medical therapies occurred. These temporal changes in clinical practice could have acted as unmeasured confounders. (5) To reduce the risk of data leakage, variables directly related to the outcome (e.g., in-hospital adverse events) were excluded from both the imputation and model-building processes. Nevertheless, because imputation was conducted on the entire dataset before splitting into training and testing subsets, some leakage during model evaluation cannot be excluded, and unrecognized leakage may remain possible given the inherent complexity of registry datasets. (6) The absence of external validation is another limitation, as the predictive performance of our models was assessed only within the current dataset and may not be fully generalizable to other populations.

## 5. Future Research Directions

Future studies should aim to integrate procedural details—including the type of stent or drug-coated balloon—along with intravascular imaging (such as minimal stent area, stent optimization, and underexpansion), as well as longitudinal clinical data, to refine risk prediction and capture dynamic changes over time. Expanding outcome definitions beyond MACE to include endpoints such as heart failure hospitalization and quality of life will provide a more comprehensive, patient-centered perspective. In addition, our study demonstrated that a collaborative approach between statisticians and cardiologists can be instrumental in identifying and interpreting modifiable risk factors. By leveraging the RF model and SHAP analysis, these explainable AI methods can be translated into practical decision-support tools integrated with electronic records, thereby facilitating clinical implementation and optimizing individualized care.

Another important direction is benchmarking ML models against established clinical risk scores. The CADILLAC risk score is particularly relevant because it incorporates key procedural and clinical factors—including age, Killip class, anemia, renal function, multivessel disease, LVEF, and final TIMI flow—to predict long-term mortality after primary PCI. Integrating CADILLAC into future analyses could clarify whether ML provides incremental prognostic value beyond this validated risk score and help refine clinical decision-making for long-term outcomes.

A further promising avenue is the integration of cardiopulmonary exercise testing (CPET) with AI-driven analytics. CPET provides well-established prognostic markers such as peak oxygen consumption (VO_2_ max), ventilatory efficiency (VE/VCO_2_ slope), and oxygen pulse, while continuous ECG monitoring during exercise can reveal dynamic changes including ST-segment shifts, arrhythmias, and conduction abnormalities. Recent advances highlight the importance of explainable AI in cardiology, particularly in applications such as ECG interpretation and arrhythmia detection [46]. By applying ML and explainable AI to these multidimensional datasets, future studies may enhance long-term prognosis prediction, identify high-risk patient subgroups, and tailor individualized rehabilitation and follow-up strategies. Such an approach could bridge interventional cardiology with cardiac rehabilitation and preventive care, thereby advancing truly patient-centered cardiovascular management.

## 6. Conclusions

In this large, real-world cohort of AMI patients, the RF model demonstrated better prognostic performance over other ML techniques, while traditional logistic regression remained competitive. Furthermore, our analysis of predictive variables revealed that among modifiable risk factors, adherence to guideline-directed optimal medical therapy, critically including DAPT, statins, and ACE inhibitors/ARBs, was a key predictor of improved long-term outcomes at both 1 year and 5 years. These findings suggest that ML approaches may provide incremental value in identifying high-risk patients, while reaffirming the significant real-world impact of optimal medical therapy on the prognosis of patients after AMI.

## Figures and Tables

**Table 1:** Baseline Characteristics of the Patients.

	1 Year			5 Years		
Characteristics	Overall	No MACE	MACE	Overall	No MACE	MACE
	*n* = 9624	*n* = 8537	*n* = 1087	*n* = 6747	*n* = 4686	*n* = 2061
**Demographics**						
Age (years)	63.5 ± 12.7	62.5 ± 12.5	71.5 ± 11.3	63.7 ± 12.7	60.7 ± 12.0	70.5 ± 11.8
Male	6912 (71.8)	6245 (73.2)	667 (61.4)	4797 (71.1)	3520 (75.1)	1277 (62.0)
BMI	24.1 ± 3.2	24.2 ± 3.2	23.2 ± 3.4	24.1 ± 3.2	24.4 ± 3.1	23.3 ± 3.3
SBP	128.3 ± 27.0	129.4 ± 26.5	119.4 ± 29.0	127.9 ± 27.1	129.6 ± 26.3	124.1 ± 28.5
DBP	78.3 ± 16.7	79.0 ± 16.4	72.8 ± 18.1	78.1 ± 16.7	79.4 ± 16.2	75.1 ± 17.4
HR	78.9 ± 19.0	78.1 ± 18.3	85.3 ± 22.8	79.2 ± 19.3	77.1 ± 17.6	84.0 ± 22.0
Clinical diagnosis						
STEMI	4337 (45.1)	3881 (45.5)	456 (42.0)	2935 (43.5)	1982 (42.3)	953 (46.2)
NSTEMI	5287 (54.9)	4656 (54.5)	631 (58.1)	3812 (56.5)	2704 (57.7)	1108 (53.8)
**Past medical history**						
Hypertension	5016 (52.1)	4313 (50.5)	703 (64.7)	3585 (53.1)	2267 (48.4)	1318 (64.0)
Diabetes mellitus	3002 (31.2)	2553 (29.9)	449 (41.3)	2138 (31.7)	1279 (27.3)	859 (41.7)
Hyperlipidemia	1523 (15.8)	1381 (16.2)	142 (13.1)	968 (14.3)	701 (15.0)	267 (13.0)
History of CAD	287 (3.0)	265 (3.1)	22 (2.0)	195 (2.9)	155 (3.3)	40 (1.9)
Current smoker	3891 (40.4)	3586 (42.0)	305 (28.1)	2682 (39.8)	2080 (44.4)	602 (29.2)
Prior MI	347 (3.6)	293 (3.4)	54 (5.0)	266 (3.9)	145 (3.1)	121 (5.9)
Prior PCI	557 (5.8)	469 (5.5)	88 (8.1)	386 (5.7)	213 (4.6)	173 (8.4)
Prior CABG	43 (0.4)	33 (0.4)	10 (0.9)	34 (0.5)	14 (0.3)	20 (1.0)
Prior CVA	694 (7.2)	553 (6.5)	141 (13.0)	522 (7.7)	275 (5.9)	247 (12.0)
CAD	52 (0.5)	42 (0.5)	10 (0.9)	31 (0.5)	15 (0.3)	16 (0.8)
A.fib	492 (5.1)	364 (4.3)	128 (11.8)	380 (5.6)	177 (3.8)	203 (9.9)
Chronic renal disease	189 (2.0)	124 (1.5)	65 (6.0)	146 (2.2)	28 (0.6)	118 (5.7)
Cancer	294 (3.1)	252 (3.0)	42 (3.9)	177 (2.6)	86 (1.8)	91 (4.4)
Chronic liver disease	82 (0.9)	73 (0.9)	9 (0.8)	56 (0.8)	38 (0.8)	18 (0.9)
COPD	235 (2.4)	190 (2.2)	45 (4.1)	173 (2.6)	87 (1.9)	86 (4.2)
**Laboratory and echocardiographic findings**						
White blood cell	14.6 ± 121.0	13.9 ± 114.3	19.8 ± 164.0	14.7 ± 105.2	14.1 ± 98.3	16.0 ± 119.4
Neutrophil	65.3 ± 92.7	64.9 ± 98.1	68.4 ± 20.5	65.2 ± 100.8	64.2 ± 119.9	67.5 ± 23.7
Hemoglobin	13.6 ± 2.1	13.7 ± 2.0	12.4 ± 2.2	13.5 ± 2.1	13.8 ± 1.9	12.7 ± 2.3
Platelet	233.0 ± 74.4	233.4 ± 73.6	230.1 ± 80.6	232.9 ± 71.9	233.9 ± 69.9	230.7 ± 76.1
HbA1c	6.6 ± 1.6	6.6 ± 1.6	6.8 ± 1.5	6.6 ± 1.6	6.6 ± 1.6	6.8 ± 1.6
Total Cholesterol	178.2 ± 43.4	179.8 ± 42.6	166.2 ± 47.5	178.3 ± 43.5	182.7 ± 41.6	168.3 ± 45.9
Triglyceride	125.4 ± 91.6	127.3 ± 93.3	110.7 ± 75.1	125.9 ± 92.9	132.7 ± 99.7	110.6 ± 72.7
HDL Cholesterol	40.7 ± 10.9	40.9 ± 10.8	39.1 ± 11.8	40.7 ± 10.9	41.2 ± 10.5	39.6 ± 11.5
LDL Cholesterol	113.8 ± 37.8	115.0 ± 37.3	104.2 ± 40.5	113.8 ± 37.8	117.2 ± 36.8	106.2 ± 38.8
hsCRP	77.2 ± 312.3	73.4 ± 323.4	107.5 ± 202.5	79.8 ± 264.8	70.8 ± 288.4	100.1 ± 199.6
Creatinine	1.2 ± 1.1	1.1 ± 0.9	1.7 ± 1.7	1.2 ± 1.1	1.0 ± 0.7	1.6 ± 1.7
eGFR	63.4 ± 24.0	65.3 ± 23.2	48.0 ± 25.2	61.8 ± 23.7	66.6 ± 21.3	50.8 ± 25.2
CK-MB (peak)	142.1 ± 677.1	130.0 ± 233.4	237.1 ± 1903.6	147.4 ± 796.2	134.1 ± 247.2	177.6 ± 1391.3
LV EF	53.1 ± 11.3	53.9 ± 10.8	46.5 ± 12.9	52.8 ± 11.6	54.7 ± 10.6	48.4 ± 12.6
**Discharge medications**						
DAPT	9026 (93.8)	8388 (98.3)	638 (58.7)	6196 (91.8)	4605 (98.3)	1591 (77.2)
SAPT	198 (2.1)	139 (1.6)	59 (5.4)	155 (2.3)	78 (1.7)	77 (3.7)
warfarin	201 (2.1)	169 (2.0)	32 (2.9)	142 (2.1)	76 (1.6)	66 (3.2)
anticoagulation	285 (3.0)	245 (2.9)	40 (3.7)	203 (3.0)	110 (2.4)	93 (4.5)
statin	8343 (86.7)	7754 (90.8)	589 (54.2)	5629 (83.4)	4213 (89.9)	1416 (68.7)
beta blocker	7604 (79.0)	7073 (82.9)	531 (48.9)	5153 (76.4)	3854 (82.2)	1299 (63.0)
ACE-inhibitor or ARB	7195 (74.8)	6688 (78.3)	507 (46.6)	5091 (75.5)	3860 (82.4)	1231 (59.7)
**Procedure details**						
Radial access	1732 (18.0)	1581 (18.5)	151 (13.9)	771 (11.4)	488 (10.4)	283 (13.7)
Multivessel disease	2911 (30.2)	2592 (30.4)	319 (29.4)	2087 (30.9)	1447 (30.9)	640 (31.1)
Left main disease	639 (6.6)	492 (5.8)	147 (13.5)	459 (6.8)	236 (5.0)	223 (10.8)
Proximal LAD lesion	4037 (41.9)	3515 (41.2)	522 (48.0)	2871 (42.6)	1902 (40.6)	969 (47.0)
Disease_extent						
1VD	4319 (44.9)	3935 (46.1)	384 (35.3)	2892 (42.9)	2157 (46.0)	735 (35.7)
2VD	3132 (32.5)	2776 (32.5)	356 (32.8)	2193 (32.5)	1499 (32.0)	694 (33.7)
3VD	2105 (21.9)	1780 (20.9)	325 (29.9)	1612(23.9)	1006 (21.5)	606 (29.4)
Complex PCI	4069 (42.3)	3595 (42.1)	474 (43.6)	2871 (42.6)	1949 (41.6)	922 (44.7)
Treated lesion extent						
1VD	7039 (73.1)	6202 (72.7)	837 (77.0)	4885 (72.4)	3355 (71.6)	1530 (74.2)
2VD	2541 (26.4)	2293 (26.9)	248 (22.8)	1826 (27.1)	1303 (27.8)	523 (25.4)
3VD	44 (0.5)	42 (0.5)	2 (0.2)	36 (0.5)	28 (0.6)	8 (0.4)
LM PCI	406 (4.2)	304 (3.6)	102 (9.4)	285 (4.2)	141 (3.0)	144 (7.0)
Graft PCI	6 (0.1)	5 (0.1)	1 (0.1)	3 (0.0)	1 (0.0)	2 (0.1)
LAD PCI	5831 (60.6)	5138 (60.2)	693 (63.8)	4115 (61.0)	2815 (60.1)	1300 (63.1)
LCX PCI	2544 (26.4)	2326 (27.3)	218 (20.1)	1775 (26.3)	1298 (27.7)	477 (23.1)
RCA PCI	3791 (39.4)	3390 (39.7)	401 (36.9)	2694 (39.9)	1906 (40.7)	788 (38.2)
Culprit LAD/LM	4947 (51.4)	4319 (50.6)	628 (57.8)	3487 (51.7)	2348 (50.1)	1139 (55.3)
Bifurcation	400 (4.2)	346 (4.1)	54 (5.0)	290 (4.3)	192 (4.1)	98 (4.8)
Ostium	390 (4.1)	337 (4.0)	53 (4.9)	288 (4.3)	192 (4.1)	96 (4.7)
Restenosis	145 (1.5)	126 (1.5)	19 (1.8)	84 (1.2)	47 (1.0)	37 (1.8)
CTO	506 (5.3)	440 (5.2)	66 (6.1)	369 (5.5)	264 (5.6)	105 (5.1)
Long stenting	1320 (13.7)	1149 (13.5)	171 (15.7)	866 (12.8)	553 (11.8)	313 (15.2)
IVUS	1944 (20.2)	1771 (20.7)	173 (15.9)	1491 (22.1)	1122 (23.9)	369 (17.9)
total stent number	1.6 ± 0.9	1.6 ± 0.9	1.6 ± 0.9	1.6 ± 0.9	1.7 ± 0.9	1.6 ± 0.8
mean stent diameter	3.2 ± 0.4	3.2 ± 0.4	3.1 ± 0.4	3.2 ± 0.4	3.2 ± 0.4	3.1 ± 0.4
total stent length	34.2 ± 20.8	34.4 ± 20.9	32.6 ± 20.1	34.4 ± 20.7	34.7 ± 21.0	33.6 ± 20.0

MACE, major adverse cardiac event; STEMI, ST-elevation myocardial infarction; NSTEMI, non-ST elevation myocardial infarction; BMI, body mass index; SBP, systolic blood pressure; DBP, diastolic blood pressure; HR, heart rate; CAD, coronary artery disease; MI, myocardial infarction; PCI, percutaneous coronary intervention; CABG, coronary artery bypass-grafting surgery; CVA, cerebrovascular attack; A.fib, atrial fibrillation; COPD, chronic obstructive pulmonary disease; HbA1c, glycated hemoglobin; HDL, high-density lipoprotein; LDL, low-density lipoprotein; hsCRP, high-sensitivity C-reactive protein; eGFR, estimated glomerular filtration rate; CK-MB, creatine kinase–myocardial band; LV EF, left ventricular ejection fraction; DAPT, dual antiplatelet therapy; SAPT, single antiplatelet therapy; ACE-inhibitor, angiotensin-converting enzyme inhibitor; ARB, angiotensin receptor blocker; LAD, left anterior descending coronary artery; VD, vessel disease; LM, left main; LCX, left circumflex coronary artery; RCA, right coronary artery; CTO, chronic total occlusion; IVUS, intravascular ultrasound.

**Table 2:** Predictions with Machine Learning Models.

5 Years	AUC	Accuracy	Precision	Recall	F1-Score
**Logistic**	0.817	0.798	0.810	0.925	0.864
**Naive Bayes**	0.772	0.755	0.790	0.883	0.834
**Elastic net**	0.817	0.794	0.790	0.958	0.866
**SVM**	0.814	0.794	0.798	0.943	0.864
**RF**	0.822	0.804	0.808	0.943	0.870
**1 Year**	**AUC**	**Accuracy**	**Precision**	**Recall**	**F1-Score**
**Logistic**	0.843	0.927	0.93	0.993	0.960
**Naive Bayes**	0.819	0.851	0.943	0.886	0.913
**Elastic net**	0.844	0.927	0.928	0.995	0.961
**SVM**	0.758	0.927	0.928	0.995	0.960
**RF**	0.847	0.930	0.928	0.998	0.962

AUC, area under the curve; logistic, logistic regression analysis; Elastic net, elastic net regression analysis; SVM, support vector machine; RF, random forest.

## Data Availability

The data presented in this study are available on request from the corresponding author.
